# Effects of dietary ratio of n-6 to n-3 polyunsaturated fatty acids on immunoglobulins, cytokines, fatty acid composition, and performance of lactating sows and suckling piglets

**DOI:** 10.1186/2049-1891-3-43

**Published:** 2012-12-27

**Authors:** Wei Yao, Jie Li, Jun jun Wang, Weiliang Zhou, Qingbo Wang, Rongchang Zhu, Fenglai Wang, Phil Thacker

**Affiliations:** 1 Ministry of Agriculture Feed Industry Centre State Key Laboratory of Animal Nutrition, China Agricultural University, Yuanming Yuan western Road, Haidian District, Beijing 100193, China; 2 Tianjin National Breeding Pig Farm, Tianjin, 301500, China; 3 Ninghe Livestock & Fisheries Bureau, Tianjin, 301500, China; 4 Department of Science, University of Saskatchewan, Saskatoon, SK S7N 5A2, Canada

**Keywords:** n-6:n-3 polyunsaturated fatty acids, Performance, Fatty acid composition, Immunoglobulin, Cytokines

## Abstract

This experiment was conducted to investigate the effects of dietary ratios of n-6:n-3 polyunsaturated fatty acids (PUFA) on the performance of lactating sows and their piglets. Thirty pregnant Landrace sows were assigned to one of three treatments from d 108 of gestation until weaning (26–29 d) and were fed diets containing different ratios of n-6:n-3 PUFA including 3:1, 9:1 and 13:1. The effects on sow and litter production traits were examined together with an assessment of sow body condition. No differences were detected among the treatments for the daily feed intake of sows or changes in sow weight and back-fat levels during lactation (*P* > 0.05). Litter size at d 14 and d 21 were tended to increase in 3:1 treatment compared with 9:1 and 13:1 treatments (*P* < 0.10). Litter weight gain (1.77 kg/d) from d 0 to d 14 was tended to increase in 9:1 groups compared with the other two treatments (*P* < 0.10). A significant difference was observed for the content of α -linolenic acid, total n-3 PUFA, and the ratio of n-6:n-3 PUFA in the colostrum, milk, and piglets plasma (*P <* 0.01). The effects of different ratios of n-6:n-3 PUFA in sow diets on colostrum, milk, and piglet plasma immunoglobulin concentrations are studied. No difference was observed among treatments in the concentrations of IgM, and IgA in colostrum (*P* > 0.05). A great significant difference for IgG concentration was observed among 3 group in colostrum. A great significant difference for IgA, and IgM (*P* < 0.01) concentrations in piglet plasma at d14 and a significant difference for IgG(*P* < 0.05) was observed at d14. Furthermore, at d 21 of lactation, piglet plasma IgG and IgA concentration were greater in 3:1 compared with 13:1 group (*P* < 0.01).

In summary, the current study demonstrated that altering the ratio of n-6:n-3 PUFA in lactating sow diet had an effect on the immune component including immunoglobulin and cytokines, and it tended to increase the litter average daily gain and improve the immune status of piglets when dietary ratio of n-6:n-3 PUFA was 9:1.

## Introduction

Essential fatty acids can be separated into two kinds of PUFA that including n-6 and n-3 PUFA, these two classes of essential fatty acids are not inter-convertible and often have important opposing physiological functions [1], the balance of essential fatty acids is very important for health and normal development.

The amount and profile of fatty acids in the diet had an effect on the lipid metabolism of animals [2]. Studies had demonstrated that animal performance could be influenced by the level or the ratio of n-6 and n-3 polyunsaturated fatty acids (PUFA) in cockerels and dogs [3,4]. The fat stores of young pig are readily mobilized under conditions of stress such as starvation Miller et al., [5]. Alterations of lipid content of the suckling pig may affect its endogenous energy supply and their ability to endure the stress of weaning [6]. Reported that adding different sources of oil including maize, linseed or tuna oils to sow diets throughout pregnancy and lactation had a subsequent effect on piglet weight 7 days post weaning. However, it is unknown whether a particular ratio of n-6:n-3 PUFA in maternal diet is beneficial to the pig performance. On the other hand, development of immune system is critical for piglet survival and normal growth. Maternally supplied fish oil altered piglet immune cell fatty acid profile and eicosanoid production [7], and n-6 and n-3 PUFA can also affect the immune response through the pathway of cyclooxygenase and lipoxygenase [8]. But whether an optimal ratio of n-6:n-3 PUFA that can improve piglet immune status is unknown.

So the aim of this study is to investigate the effect of dietary ratios of n-6:n-3 PUFA on the immune component, fatty acid composition, and the performance of lactating sows and their suckling piglets.

## Materials and methods

### Animals and experimental design

Thirty multiparous sows (Landrace) with an initial body weight of 248.26 ± 9.04 kg, and parity is 4.4 ± 0.78 were selected from the herd at the Tianjin Pig Breeding Farm (Tianjin, China) and assigned to three treatments comprised of diets containing different ratios of n-6:n-3 PUFA including 3:1, 9:1 and 13:1 taking into account parity, body weight and expected farrowing date. The number of sows in 3:1, 9:1, and 13:1 group is 12 per treatments at the beginning of the trial, finally was 9, 10, and 11 because of the disease, respectively. All animal procedures were approved by the Institutional Animal Care and Use Committee of China Agricultural University (Beijing, China).

This experiment was completed during a 5-week period from the July to September. The sows were housed individually in 2.2 x 1.8 m farrowing crates with solid concrete floors combined with slats of iron. Heat lamps were installed for the first week after birth,and after one week of age it was used only in night. slatter floor were used in all pens. To guarantee that all sows nursed a similar number of piglets (about 10 piglets per sow), litter sizes were adjusted by cross-fostering piglets within 24 h of birth. Piglets received an intramuscular injection of iron dextran 3 days after birth.

The experimental diets were provided from d 108 of gestation until weaning (26–29 d). During the suckling period, all sows were fed four times each day in an attempt to maximize feed intake. Piglets were provided with creep feed from two weeks of age. The sow diets (Table 1) were based on corn-soybean meal and were formulated to meet or exceed the requirements for all nutrients [9]. The digestible energy level was designed at 3.39Mcal/kg according to the guide. The ingredients in the formula were almost the same in 3 treatment except the ratio of n-6:n-3 PUFA was modulated by changing the percentage of corn oil and linseed oil in a mixed oil supplement which was mixed into the diet at a level of 4.0%. We analyzed the fatty acid composition in the corn, the corn oil, and linseed oil, so we can caculate the content of these two oils in these three diets. 2% linseed oil and 0.8% corn oil were used in 3:1 treatment, 1.4% linseed oil and 2.6% corn oil were used in 9:1 treatment, 0.8% linseed oil and 3.2% corn oil were used in 13:1 treatment. The corn oil was obtained from a local super market and linseed oil was obtained from a commercial company (Hong Jin Yuan, Neimeng, China). The fatty acid composition of the experimental diets was shown in Table 2.

**Table 1:** Ingredient and chemical composition of experimental diets

**Ingredient (% as fed)**	**Dietary n-6:n-3 PUFA ratio**		
	**3:1**	**9:1**	**13:1**
Corn	58.75	58.75	58.75
Soybean meal (45% CP)	27.25	27.25	27.25
Wheat bran	4.00	4.00	4.00
Fish meal (60% CP)	2.00	2.00	2.00
Limestone	1.64	1.64	1.64
Oil mixture^a^	4.00	4.00	4.00
Salt	0.60	0.60	0.60
Dicalcium phosphate	0.84	0.84	0.84
L-lysine (65%)	0.12	0.12	0.12
Vitamin and mineral premix^b^	0.80	0.80	0.80
Analyzed chemical composition (% as fed)			
Dry matter	89.26	90.60	89.35
Crude protein	18.13	18.31	18.31
Lysine	0.96	0.92	1.08
Calcium	1.02	0.98	0.95
Phosphorus	0.58	0.57	0.56

^a^Oil was a mixture of different combinations of corn and linseed oil. 3.2% linseed oil and 0.8% corn oil in 3:1 treatment, 1.4% linseed oil and 2.6 corn oil in 9:1 treatment, 0.8% linseed oil and 3.2% corn oil in 13:1 treatment.

^b^Composition per kilogram of diet:Vitamin A, 12,000 IU; Vitamin D_3_, 2,000 IU; Vitamin E, 24 IU; Vitamin K_3_, 2.0 mg; Vitamin B_1_, 2.0 mg; Vitamin B_2_, 60 mg; Vitamin B_6_, 4.0 mg; Vitamin B_12_, 24 μg; niacin, 30 mg; pantothenic acid, 20 mg; folic acid, 3.6 mg; biotin, 0.40 mg; choline, 400 mg; Fe, 96 mg; Cu, 8.0 mg; Zn, 120 mg; Mn, 40 mg; I, 0.56 mg; Se, 0.40 mg; phytase, 120 mg.

**Table 2:** Fatty acid composition of the experimental diets (mg/g of diet)

**Item**	**Dietary n-6:n-3 PUFA ratio**		
	**3:1**	**9:1**	**13:1**
C14:0	0.06	0.03	0.04
C16:0	5.34	7.65	7.67
C18:0	1.28	1.37	1.36
SFA	6.68	9.04	9.06
C16:1n-7	0.08	0.09	0.09
C18:1n-9	10.05	13.77	13.85
C18:1n-7	0.39	0.45	0.43
MUFA	10.52	14.31	11.13
C18:2n-6	15.94	25.14	24.92
n-6 PUFA	15.94	25.14	24.92
C18:3n-3	5.37	2.71	1.91
C20:5n-3	0.03	0.03	0.03
C22:6n-3	0.03	0.04	0.03
n-3 PUFA	5.43	2.78	1.97
Ratio of n-6:n-3 PUFA	2.93	9.04	12.65

### Data recording and sample collection

Sows were weighed at day 107 of gestation and at weaning (26–29 d) and back fat thickness at the 10th rib (65 mm from the midline) was measured at the day of farrowing and at weaning using an A-mode Ultrasound (Renco Lean Meter, Renco, Minneapolis, MN). Individual piglets were weighed within the first 24 h of farrowing as well as at d 14 and weaning, and the litter size at these times were also recorded. Sow feed intake was recorded daily. Colostrum (20 mL) was collected 0.5 h before the birth of first piglet from the functional glands of each sow before suckling, and milk (10 mL) was collected from sows 1 h later in 10:00 at d 21 of lactation after the injection of 20 IU oxytocin (Qilu limited company, Shandong, China). Colostrum and milk samples were immediately frozen at −20°C for later analysis. Piglet blood samples were collected from the anterior vena cava at d 14 and 21 of lactation (six piglets per treatment, 3 male and 3 female) into 5-mL heparinized vacutainer tubes and centrifuged (3500 × g for 10 min, Ciji 800 Model Centrifuge, Surgical Instrument Factory, Changzhou, China) for plasma extraction. Aliquots of plasma samples were stored at −80°C until analysis.

### Analytical methods

Samples of diets were analyzed for dry matter, crude protein, lysine, calcium and total phosphorus using [10] procedures. Lysine was analyzed using ion-exchange chromatography with an Automatic Amino Acid Analyser (L-8800 Hitachi Automatic Amino Acid Analyzer, Tokyo, Japan) after hydrolyzing with 6 M HCl at 110°C for 24 h.

The fatty acid composition of diets, plasma and milk were analyzed by gas chromatography. Lipids were extracted by chloroform: methanol (1:1) as described by [11]. Fatty acid methyl esters were prepared using a mixture of potassium hydroxide and methanol (0.4 mol.L^-1^). A gas chromatograph (Hewlett Packard Avondale, PA) equipped with a 1177 injector, a flame ionization detector and a 100 m x 250 μm x 0.25 μm CP-Sil 88 Capillary Chromatographic Column (Varian, Palo Alto CA) specific for fatty acid methyl esters was used. The injector and detector temperatures were kept at 250°C. Helium was used as the carrier gas with a flow rate of 1.0 mL min^-1^ and a split ratio of 1:40. The column temperature was programmed to be 180°C at the start and was increased to 215°C at a rate of 10°C min^-1^ and then held constant for 17 min. The total analysis time was 45 min. The fatty acids were identified by comparing the retention times of the peaks with those of known standards (Sigma, St Louis, MO). Saturated fatty acids were recorded as the sum of C14:0, C16:0, and C18:0. The monounsaturated fatty acids included C16:1n-7 and C18:1n-9. The n-3 PUFA were recorded as the sum of C18:3(n-3), C20:5(n-3), C22:5(n-3), and C22:6(n-3) while the n-6 PUFA were recorded as the sum of C18:2(n-6) and C20:4(n-6).

Immunoglobulin including piglet plasma, colostrumn, and milk concentrations of IgG, IgA, and IgM were assayed using specific pig-ELISA kits (R & D Incorporated, Minneapolis, MN) run according to the manufacturer’s instructions. Piglet plasma cytokines including interleukin-1β(IL-1β), IL-6, and tumor necrosis factor-α (TNF-α) were assayed using specific pig-ELISA kits (R&D Incorporated, Minneapolis, MN) run according to the manufacturer’s instructions. The color change was measured spectrophotometrically at a wavelength of 450 nm and the sample concentrations were determined by comparing the optical density of the samples to a standard curve.

### Statistical analyses

Performance data were analyzed using the GLM procedure of the SAS program (SAS Institute Inc., Cary, NC). The parity of sows was included as a covariate in the model to evaluate its effects on reproductive performance. Lactation duration was also included as a covariate in the model to evaluate its effect on litter weight at weaning (d 26–29). A one-way ANOVA was used followed by Duncan’s Multiple Range Test when significant differences were obtained. Sow and latter litter was considered as the experimental unit in the analysis of reproductive performance, immunoglobulin titers, fatty acid composition and serum metabolites and the pig for litter serum immune components and fatty acid composition. A value of *P* < 0.05 was used to indicate statistical significance.

## Results

### Sow and piglet performance

The effect of dietary ratios of n-6:n-3 PUFA on performance of sows and their suckling piglets was presented in Table 3. No differences were observed among treatments for sow average daily feed intake, body weight loss, and change in back fat thickness during lactation (not shown in statistical), the litter size at birth and d 28 were unaffected by treatments (*P* > 0.05), the litter size at d 14 and d 21 of lactation in 3:1 group was tended to increase compared with 9:1 and 13:1 groups (*P* < 0.10). Litter weight at birth, d 14, and d 28 of lactation were also unaffected by treatment (*P* > 0.05), but litter average daily gain from d 0 to d 14 was tended to increased in 9:1 treatment compared with the other two treatments (*P* < 0.10). The mortality during lactation seems to be least in 3:1 treatment.

**Table 3:** Effect of dietary ratio of n-6:n-3 PUFA on sow and piglets performance

**Item**	**Dietary n-6:n-3 PUFA ratio**			**SEM**	**P**
	**3:1**	**9:1**	**13:1**		
Number of sows	9	10	11	-	-
Parity	4.33	4.30	4.54	0.49	0.98
Pigs/litter					
Birth	10.31	9.47	9.68	0.43	0.58
14 d	9.75	9.06	8.14	0.40	0.09
21 d	9.75	8.86	8.05	0.39	0.06
28 d	9.30	8.85	8.07	0.30	0.11
Litter weight (kg)					
At birth	15.08	12.26	13.45	0.55	0.15
14 d	36.26	36.88	32.55	0.93	0.47
28 d	66.93	72.20	63.83	1.20	0.43
Litter weight gain (kg/d)					
D 0 to d 14	1.52	1.77	1.35	0.21	0.10
D 0 to d 28	2.27	2.08	2.02	0.20	0.41

*The means were covariately adjusted, within a row, treatments with unlike superscripts are different (*P* < 0.05).

### Effect of maternal diet on fatty acid composition in colostrum, milk, and plasma

The fatty acid composition of the colostrum is presented in Table 4. Saturated fatty acids (SFA), Monounsaturated fatty acids (MUFA), and n-6 PUFA were unaffected by treatment (*P* > 0.05). Significant decreases were observed for the content of linolenic acid, total n-3 PUFA, and the ratio of total n-3: n-6 PUFA (*P* < 0.01) as the dietary ratio of n-6:n-3 PUFA widened. The fatty acid composition of milk at d 14 of lactation is shown in Table 5. SFA and n-6 PUFA were unaffected by treatments (*P* > 0.05), but the amounts of MUFA including C18:1n-9 and C18:1n-7 were greater in 3:1 and 9:1 groups compared with 13:1 group (*P* < 0.05). The contents of α-linolenic acid and eicosapentaenoic acid (EPA) in 3:1 treatment were greater compared with the other two treatments (*P* < 0.05), and the ratio of n-3:n-6 PUFA significantly decreased as the dietary ratio of n-6:n-3 PUFA increased from 3:1 to 13:1 (*P* < 0.01). Effects of dietary ratio of n-6:n-3 PUFA on the fatty acid composition of piglet plasma at d 21 of lactation was shown in Table 6. No significant difference was observed for SFA, MUFA, and n-6 PUFA except C18:0 (*P* < 0.05). The content of α-linolenic acid (*P* < 0.05), docosahexaenoic acid (P < 0.01) were greater in 3:1 group compared with 9:1 and 13:1 groups, and the ratio of n-6:n-3 PUFA increased as the ratio of dietary n-6:n-3 PUFA wilden from 3:1 to 13:1.

**Table 4:** Effects of different ratios of n-6:n-3 PUFA in the sow diet on the fatty acid composition of colostrum

**Item**	**Dietary n-6:n-3 PUFA ratio**			**SEM**	**P**
	**3:1**	**9:1**	**13:1**		
Fatty acid composition (mg/g)					
C14:0	0.51	0.52	0.41	0.10	0.71
C16:0	6.99	7.46	6.46	0.94	0.76
C18:0	1.99	2.02	1.65	0.53	0.86
SFA	9.50	9.99	8.51	1.42	0.76
C16:1n-7	0.71	0.72	0.63	0.17	0.92
C18:1n-9	8.72	9.74	9.12	1.06	0.79
C18:1n-7	0.65	0.73	0.66	0.08	0.72
MUFA	10.08	11.20	10.40	1.20	0.80
C18:2n-6	10.89	11.43	11.90	1.32	0.87
C20:4n-6	0.33	0.32	0.35	0.17	0.95
N-6 PUFA	11.23	11.75	12.25	1.52	0.89
C18:3n-3	1.75^a^	1.03^b^	0.88^b^	0.14	< 0.01
C20:5n-3	0.06	0.05	0.04	0.01	0.32
C22:6n-3	0.05	0.04	0.04	0.01	0.72
N-3 PUFA	1.85^a^	1.12^b^	0.95^b^	0.16	< 0.01
Ratio of n6:n3 PUFA	5.93^a^	10.56^b^	13.84^b^	1.22	< 0.01

*Within a row, treatments with unlike superscripts are different (*P* < 0.05).

**Table 5:** Effects of different ratios of n-6:n-3 PUFA in the sow diet on the fatty acid composition of milk at d 21 of lactation

**Item**	**Dietary n-6:n-3 PUFA ratio**			**SEM**	**P**
	**3:1**	**9:1**	**13:1**		
Fatty acid composition (mg/g)					
C14:0	1.86	2.04	1.93	0.25	0.88
C16:0	17.30	18.39	17.25	2.22	0.92
C18:0	2.77	2.55	2.34	0.30	0.60
SFA	21.94	22.98	21.53	2.66	0.92
C16:1n-7	4.46	4.40	3.64	0.67	0.64
C18:1n-9	15.82^a^	15.83^a^	11.89^b^	1.05	0.03
C18:1n-7	0.92^ab^	1.04^aa^	0.62^bb^	0.10	0.04
MUFA	21.20	21.26	16.16	1.58	0.07
C18:2n-6	12.84	11.60	12.06	1.65	0.87
C20:4n-6	0.24	0.27	0.24	0.03	0.78
N-6 PUFA	13.08	11.87	12.30	1.68	0.88
C18:3n-3	3.97^a^	1.08^b^	1.00^b^	0.58	< 0.01
C20:5n-3	0.07^a^	0.03^b^	0.04^b^	0.01	0.02
C22:6n-3	0.04	0.04	0.04	0.01	0.74
N-3 PUFA	4.08^a^	1.15^b^	1.07^b^	0.58	< 0.01
Ratio of n-6:n-3 PUFA	3.21^a^	10.33^b^	11.48^c^	0.33	< 0.01

*Within a row, treatments with unlike superscripts are different (*P* < 0.05).

**Table 6:** Effects of dietary ratio of n-6:n-3 PUFA on the fatty acid composition of piglet plasma at d 21 of lactation

**Item**	**Dietary n-6:n-3 PUFA**			**SEM**	***P***
	**3:1**	**9:1**	**13:1**		
Fatty acid composition, mg/g					
C14:0	0.02	0.02	0.02	0.004	0.71
C16:0	0.70	0.46	0.47	0.11	0.28
C18:0	0.39^a^	0.22^b^	0.22^b^	0.05	0.04
SFA	1.11	0.70	0.71	0.16	0.17
C16:1	0.07	0.05	0.06	0.01	0.83
C18:1n-9	0.34	0.23	0.22	0.06	0.36
C18:1n-7	0.04	0.03	0.03	0.007	0.48
MUFA	0.44	0.32	0.30	0.08	0.43
C18:2n-6	0.48	0.49	0.60	0.05	0.22
C20:4n-6	0.12	0.11	0.12	0.03	0.96
N-6 PUFA	0.59	0.6	0.71	0.07	0.39
C18:3n-3	0.05^a^	0.02^b^	0.02^b^	0.005	<0.01
C20:5n-3	-	-	-	-	-
C22:6n-3	0.04^a^	0.03^b^	0.03^b^	0.003	0.04
N-3 PUFA	0.09^a^	0.04^b^	0.05^b^	0.02	<0.01
Ratio of n-6:n-3 PUFA	6.78^a^	13.57^b^	15.49^c^	0.72	<0.01

*Within a row, treatments with unlike superscripts are different (*P* < 0.05).

### Effect of maternal diet on the concentrations of immunoglobulin (IgM, IgG and IgA)

The effects of different ratios of n-6:n-3 PUFA in sow diets on colostrum, milk, and piglet plasma immunoglobulin concentrations are presented in Table 7. No difference was observed among treatments in the concentrations of IgM, and IgA in colostrum (*P* > 0.05). A great significant difference for IgG concentration was observed among 3 group in colostrum. A great significant difference for IgA, and IgM (*P* < 0.01) concentrations in piglet plasma at d14 and a significant difference for IgG (*P* < 0.05) was observed at d14. Furthermore, at d 21 of lactation, piglet plasma IgG and IgA concentration were greater in 3:1 compared with 13:1 group (*P* < 0.01).

**Table 7:** Effects of different ratios of n-6:n-3 polyunsaturated fatty acids in the sow diet on the concentrations of IgM, IgG, and IgA in milk and piglet plasma

**Item, mg/ml**	**Dietary n-6:n-3 PUFA ratio**			**SEM**	**P**
	**3:1**	**9:1**	**13:1**		
Colsotrum					
IgM	3.22	4.18	4.21	0.63	0.47
IgG	27.02^a^	41.14^b^	36.30^b^	2.82	< 0.01
IgA	7.52	9.55	8.23	0.84	0.25
Milk					
IgM	0.95^a^	1.34^b^	1.22^ab^	0.10	0.05
IgG	0.32	0.36	0.39	0.02	0.14
IgA	3.37	3.46	3.23	0.38	0.92
Piglet plasma at d 14 of lactation					
IgM	0.48^a^	0.41^b^	0.50^a^	0.02	0.01
IgG	6.11^a^	5.33^b^	6.81^c^	0.19	0.02
IgA	0.20^a^	0.15^b^	0.17^b^	0.01	< 0.01
Piglet plasma at d 21 of lactation					
IgM	1.20^ab^	1.22^a^	1.06^b^	0.05	0.07
IgG	6.78^a^	6.85^a^	5.68^b^	0.23	0.01
IgA	0.21^a^	0.18^ab^	0.17^b^	0.01	0.05

*Within a row, treatments with unlike superscripts are significantly different (*P* < 0.05).

### Effect of maternal diet on the concentrations of cytokines (interleukin-1β, interleukin-2, and tumor necrosis factor-α

A significant difference was observed for IL-1β (interleukin-1β) among treatments at d 14 of lactation in Table 8, as well as the value of IL-1β was the smallest in 9:1 group (*P* < 0.01), and the content of tumor necrosis factor-α (TNF-α) was also tended to decrease in 9:1 group (*P* < 0.10) at d 21 of lactation.

**Table 8:** Effects of different ratios of n-6:n-3 polyunsaturated fatty acids in the sow diet on the concentrations of IL-1β, IL-6, and TNF-α in piglet plasma

**Item, ng/L**	**Dietary n-6:n-3 PUFA ratio**			**SEM**	***P***
	**3:1**	**9:1**	**13:1**		
Piglet plasma at d 14 of lactation					
IL-1β	831.19^a^	612.98^b^	710.83^b^	38.08	< 0.01
IL-6	84.49	83.33	80.56	3.14	0.67
TNF-α	163.00	182.45	158.73	17.08	0.59
Piglet plasma at d 21 of lactation					
IL-1β	742.26	652.38	740.71	57.75	0.47
IL-6	85.65	86.16	90.83	3.91	0.60
TNF-α	212.18	153.64	178.73	15.65	0.06

*Within a row, treatments with unlike superscripts are significantly different (*P* < 0.05).

## Discussion

The present experiment showed that the effect of dietary ratios of n-6:n-3 PUFA on the sow and progeny performance, fatty acid composition, and immune component including immunoglobulin and cytokines under conditions of the oil content in the diet is 4.0%.

The content of fat and energy in milk played an important role in keeping piglet alive [12], and the improvement in litter weight by adding fat to sow diets had been observed previously [13,14]. In this study, altering maternal dietary ratio of n-6:n-3 PUFA was tended to change the litter average daily gain from d 0 to d 14, and it was greater in 9:1 group compared with the other two groups, but no difference was observed for other performance criterias, some potential effect and the number of sows need to be considered [6]. Reported that offering maize, linseed or tuna oils throughout pregnancy and lactation on sows, the ratio of n-6:n-3 PUFA in maize oil, tuna oil, and linseed oil treatments were 17.4, 3.4, and 3.5, respectively. Piglets suckling maize or tuna oil sows were heavier than piglets suckling linseed oil sows 7 days post weaning. Edwards and Pike (1997) showed that pregnancy length were increased by offering sows n-3 PUFA as fish meal, and piglet born to sows offered n-3 PUFA may have been born more prematurely and less well prepared for birth. But in our experiment. The pregnancy length seems not be affected by the treatment. However, [15] had demonstrated that an inadequate supply of n-3 PUFA to the pre-term babies is associated with impairment of visual acuity and cognitive development, since commercial sow diets were based on cereals which are lacked of n-3 PUFA, the piglet viability and their growth during lactation may be affected by the imbalance or deficiency of n-3 PUFA. And on the other side, the number of sows should be considered to determine the effect of the ratio of n-6:n-3 PUFA on the performance of lactating sows and their piglets.

Via desaturase-elongase pathway, some long-chain PUFA can be converted in to the linoleate and alpa-linolenate, the precuros of essential fatty acid [16]. Among long-chain PUFA, arachidonic aicd is of particular importance because it is the major regulators of intestinal homeostasis and repair following injury, gastrointestial disturbances rank among the leading cause of neonatal morbidity, and mortality [17]. And research showed that desaturation to ^13^C-18:3(N-6) increased linearly in pigs fed arachidonate but the alternate elongation to ^13^C-20:2(n-6) was markedly elevated in pigs fed 0% arachinonate [17]. And on the other hand. Hepatic flux decreased with postnatal age, wherea intestinal flux did not change. In our study, the desaturase-elongase pathway were not analyzed, but the piglet mortaliy during lactation in 3:1 was the least. Additionally, the arachidonic acid content was similar in piglet plasma. It seems the content of precusor in diet and the age of piglets was critical to the effect of those fatty acids.

Colostrum is characterized by a high concentration of IgG and lower concentrations of IgA and IgM [18]. Passive immunity or a sufficient intake of colostral immunoglobulins by piglets plays an important role in the early life [19]. In this study, IgG concentration was highest in 9:1 treatments compared with the other two treatments, and the average daily gain in 9:1 group was also tended to increase. IgM concentration in milk was greater in 9:1 treatment among treatments.This result was consisted with the previous study [20]. Rooke and Bland (2002) reported that IgG synthesis by piglets is positively correlated to the amount of maternal IgG absorbed, thus reinforcing the importance of an adequate IgG intake from colostrum. A highest value of IgG concentration was observed in piglet plasma at d 21 of lactation, which is positively related with the amount of IgG in colostrum [21]. However, there is no clear pattern between the change of immunoglobulin concentrations in piglet plasma and the ratio of n-6: n-3 PUFA. At this time, it is unclear the mechanism that involved n-6 and n-3 PUFA in changing the IgG, IgA, and IgM concentrations. A possible explanation is that n-6 and n-3 PUFA are involved in IL production. Interleukins, as well as isotype specific lymphokines, play an important role in regulation of immunoglobulin synthesis in mouse [22]. IL-2 increased the production of IgG, IgA, and IgM, as reported by [23]. Endres S et al. [24] reported that the decreased production of interleukin-1 and tumor necrosis factor was accompanied by a decreased ratio of arachidonic acid to EPA in the membrane phospholipids of mononuclear cells. On the other hand, eicosapentaenoic acid, a kind of n-3 PUFA, in membranes competes with arachidonic acids as substrates for cyclooxygenase and lipoxygenase enzymes, decreasing the production of arachidonic acid-derived eicosanoids such as prostaglandin E_2_, which can affect the production of immunoglobulin directly [25]. Changes in dietary n-6: n-3 PUFA ratios can induce significant alterations in the composition and function of immune cell membranes [26,27], these can possibly explain the potential effects of ratio of n-6: n-3 on the immunoglobulin.

Cytokines play an important role in immunoregulation. IL-1β, IL-6, and TNF-α is among the most important cytokines produced by monocytes and macrophages. These cytokines can mediate the systemic effects of inflammation such as fever, loss of appetite, mobilization of protein and fat, and acute protein synthesis [28]. Inappropriate amounts or overproduction can be dangerous, these cytokines, especially TNF-α, are implicated in causing some of the pathological responses that occur in inflammatory conditions or some inflammation-associated diseases [29]. An imbalance of the membrane contents of n-6 and n-3 PUFA resulting from dietary deficiency, aging, and diseases may cause abnormalities in neurotransmission and inflammatory responses by changing membrane fluidity, lipid peroxidation, eicosanoid production [30]. In this study, piglet plasma IL-1β concentration were decreased in 9:1 group at d 14 of lactation, and TNF-α was also tended to decrease in this group. It seems the piglet inflammation prevention was improved when dietary ratio of n-6: n-3 was 9:1. Indeed, an increased n-6: n-3 PUFA ratio and decreased n-3 fatty acid fatty acids concentration have been found in patients with inflammatory disorders [31]. Cai S et al. [32] reported that EPA attenuated IL-1β-induced behavioral changes, and γ-linolenic acid reduced hippocampal PGE_2_ concentration in rats given IL-1β. It suggested that EPA, γ-linolenic acid, and arachidonic acid play different roles in the neuroendocrine-immune network.

In summary, our study demonstrated that ratio of n-6:n-3 PUFA in plasma, colostrum, milk, and immune components including immunoglobulin and cytokines were well affected by altering the ratio of n-6:n-3 PUFA in lactating sow diets, and it was tended to increase the litter average daily gain from d 0 to d 14 when dietary ratio of n-6: n-3 was 9:1. Further studies might examine whether there is an interaction between dietary energy level and the ratio of n-6: n-3 on performance of sows and their suckling piglets.

## Competing interests

All authors declare that they have no competing interests.

## Authors’ contribution

WY and JL carried out the experimental design and the whole experimental implement including chemical analysis, data collecting and analysis and the writing of this article. JjW carried out checking the article. WZ, QW, RZ carryied out supplying the experimental animals and place. FW was the tutor, carryied out the design of this experiment and check of this article. PT carried out checking the article. All authors read and approved the final manuscript.
